# The genetic architecture of the MHC class II region in British Texel sheep

**DOI:** 10.1007/s00251-016-0962-6

**Published:** 2016-12-05

**Authors:** Alsagher O. A. Ali, Abigail Stear, Karen Fairlie-Clarke, Gholamreza Nikbakht Brujeni, N. Mahiza Md Isa, M. Shahrom Bin Salisi, Katarzyna Donskow-Łysoniewska, David Groth, Johannes Buitkamp, Michael J. Stear

**Affiliations:** 1 Animal Medicine Department, Faculty of Veterinary Medicine, South Valley University, Qena, Egypt; 2 Institute of Biodiversity, Animal Health and Comparative Medicine, University of Glasgow, Garscube Campus, Bearsden Road, Glasgow, Scotland G61 1QH UK; 3 Department of Microbiology and Immunology, Faculty of Veterinary Medicine, University of Tehran, Tehran, Iran; 4 Faculty of Veterinary Medicine, Universiti Putra Malaysia, UPM, 43400 Serdang, Selangor Darul Ehsan Malaysia; 5 Department of Parasitology, Institute of Zoology, Faculty of Biology, University of Warsaw, ul. Miecznikowa, 02-096 Warsaw, Poland; 6 School of Biomedical Sciences, CHIRI Biosciences Research Precinct, Faculty of Health Sciences, Curtin University, GPO Box U1987, Perth, WA 6845 Australia; 7 Bavarian State Research Center for Agriculture, Institute of Animal, Breeding, 85580 Grub, Germany; 8 Department of Animal, Plant and Soil Science, Agribio, La Trobe University, Bundoora, VIC 3086 Australia

**Keywords:** Sheep, Texel breed, Major histocompatibility complex, Class II region, Polymorphism

## Abstract

Understanding the structure of the major histocompatibility complex, especially the number and frequency of alleles, loci and haplotypes, is crucial for efficient investigation of the way in which the MHC influences susceptibility to disease. Nematode infection is one of the most important diseases suffered by sheep, and the class II region has been repeatedly associated with differences in susceptibility and resistance to infection. Texel sheep are widely used in many different countries and are relatively resistant to infection. This study determined the number and frequency of MHC class II genes in a small flock of Texel sheep. There were 18 alleles at *DRB1*, 9 alleles at *DQA1*, 13 alleles at *DQB1*, 8 alleles at *DQA2* and 16 alleles at *DQB2*. Several haplotypes had no detectable gene products at *DQA1*, *DQB1* or *DQB2*, and these were defined as null alleles. Despite the large numbers of alleles, there were only 21 distinct haplotypes in the population. The relatively small number of observed haplotypes will simplify finding disease associations because common haplotypes provide more statistical power but complicate the discrimination of causative mutations from linked marker loci.

**Electronic supplementary material:**

The online version of this article (doi:10.1007/s00251-016-0962-6) contains supplementary material, which is available to authorized users.

## Introduction

The major histocompatibility complex is possibly the most important genetic system for disease resistance because it influences resistance to a wide variety of infectious, parasitic and autoimmune diseases. Also, it is often amongst the genetic regions that account for the largest amount of variation in disease susceptibility. Consequently, there has been considerable effort devoted to defining genetic variation at the MHC in sheep (Ballingall et al. 2008; Dukkipati et al. 2006; Herrmann et al. 2005; Hickford et al. 2004b; Nikbakht et al. 2012; Scott et al. 1992; Snibson et al. 1998). Multiple alleles have been identified, especially at the *DRB1* locus, and a standard nomenclature has been developed (https://www.ebi.ac.uk/ipd/mhc/).

The next stage in defining the ovine MHC and using it to understand and control disease is to determine how the alleles are arranged into haplotypes and the frequency of these haplotypes. The definition of haplotypes and their frequency is necessary to understand the genetic architecture of the MHC and to design and interpret powerful analyses of the way the MHC influences susceptibility and resistance to the major diseases of sheep. For example, the power of statistical analyses to detect associations between MHC alleles and disease depends in part upon allele and haplotype frequencies (Stear et al. 2007), whilst the ability to discriminate between markers and causative mutations depends upon the extent of linkage disequilibrium. When linkage disequilibrium is high, alleles at different loci often occur together in the same animal and the effects of the different loci cannot be easily disentangled.

One of the best examples of an association between the MHC and a major disease of livestock is nematode infection in sheep (Hassan et al. 2011; Stear et al. 2005). Texel sheep are widely used as sires to create crossbred lambs, and they are relatively resistant to nematode infection (Sayers et al. 2005). Therefore, this research describes the alleles and haplotypes at the class II MHC region in a flock of Texel sheep.

## Materials and methods

### Sheep

Three consecutive cohorts of lambs were studied to give a total of 235 animals. They came from a small flock maintained at Blythbank farm in the Scottish Borders by the Roslin Institute and have been described previously (Bishop et al. 2004). The flock was created by grading up a small flock of Texel-Oxford ewes as well as purchasing purebred Texel ewes. Sires were all purebred Texel donated by the Texel Sire Reference Society, purchased or homebred. Blood samples were collected by jugular venipuncture into a vacutainer tube (Becton-Dickinson) containing EDTA as an anticoagulant. Buffy coat and plasma were separated by centrifugation at 1200*g* for 20 min and stored at −20 °C until required. Genomic DNA was extracted from the buffy coat using QIAamp DNA Blood Maxi Kits (Qiagen) following the manufacturer’s instructions.

### Amplification and sequencing

In sheep, the MHC region has been split by an inversion on chromosome 20 (Lee et al. 2012). The classical antigen-presenting loci occur in the class IIa region closely linked to the class I region (Siva Subramaniam et al. 2015). This study focuses on the class IIa region.

#### Ovar DRB1

Primers ERB3 and SRB3 (Table 1) (Konnai et al. 2003) were used to amplify the exon 2 region of the *DRB1* locus. Primers DRB1 275 and DRB1 268 (Table 1) located within intron 1 and intron 2, respectively, were used to obtain complete exon 2 sequences (Ballingall et al. 2008). Each PCR reaction was carried out in a final volume of 20 μl containing 50 ng of genomic DNA, 0.5 μM of each primer, 250 μM dNTP (Invitrogen), 1 unit Taq DNA polymerase (Qiagen), 1× reaction buffer (supplied) and 3.375 mM MgCl_2_. Amplification was carried out in a Duocycler Thermocycler (VWR International Ltd., UK) and for DRB1 275 and DRB1 268 consisted of denaturation at 95 °C for 2 min, followed by 32 cycles of 95 °C for 60 s, 60 °C for 60 s and 72 °C for 60 s; this was followed by an extension step at 72 °C for 5 min. For primer pair ERB3 and SRB3, denaturation at 94 °C for 2 min, followed by 32 cycles of 94 °C for 30 s, 61 °C for 30 s and 72 °C for 30 s followed by an extension step at 72 °C for 5 min was performed.

**Table 1:** The primers used to amplify MHC class II loci

Primer	Sequence	Target	Size (bp)
*DRB1*			
275 (F)	ATTAGCCTCTCCCCAGGAGTC	Intron 1	368
268 (R)	CACACACACACTGCTCCACA	Intron 2	
ERB3 (F)	CTCTCTCTGCAGCACATTTCCT	Exon 2	276
SRB3 (R)	CGCTGCACAGTGAAACTC	Exon 2	
*DQA1*			
NikDQA1 (F)	ACTGGCCACAAATGAAGCCCACAA	Intron1	525
NikDQA1 (R)	AGAAGGCAGAAGATGAGGGTTCAG	Intron 2	
92.y085 (F)	CTCCGACTCAGCTGACCA	Exon2 and flanking intron 1	268
Z28518 (R)	AACACTTACTGTTGGTAGCAGCA	Exon2 and flanking intron 2	
Z28518 (F)	CCCTGACTCAGCTGACCACA	Exon2 and flanking intron 1	268
*DQA2*			
DQA2s-up (F)	ACTACCAATCTCATGGTCCCTCT	Exon 2	241
DQA2s-dn (R)	GGAGTAGAATGGTGGACACTTACC	Exon 2	
*DQB1*			
JM05	TCTCCCCGCAGAGGATTTCGTG	Exon2 and flanking intron 1	278
JM06	CTCGCCGCTGCCAGGTGAAGG	Exon2 and flanking intron 2	
Lfl#991	CTGACCGAGCGGCTGT	Intron 1	405
Lfl#994	CGGCTCTCTGTCCCATCC	Intron 2	
*DQB2*			
JM05	TCTCCCCGCAGAGGATTTCGTG	Exon2 and flanking intron 1	277
JM07	GCCGCTGCAAGGAGGTGATGAG	Exon2 and flanking intron 1	
JM05mjs	TCCCCGCAGAGGATTTCCTG	Exon2 and flanking intron 1	

#### Ovar DQA1

Primers NikDQA1F and NikDQA1R (Table 1) were designed to amplify the exon 2 region of the *DQA1* locus. Specific primers were also designed to detect alleles LN736359 and Z28518 (92.y085F and Z28518R; Z28518F and Z28518R, respectively) (Table 1). Each PCR reaction was carried out in a final volume of 20 μl containing 50 ng of genomic DNA, 0.5 μM of each primer, 250 μM dNTP (Invitrogen), 1 unit Taq DNA polymerase (Qiagen) and 1× reaction buffer containing 1.5 mM MgCl_2_ (supplied). Amplification for all primer pairs was denaturation at 95 °C for 2 min followed by 30 cycles of 95 °C for 30 s, 62.2 °C for 30 s and 72 °C for 60 s followed by an extension step at 72 °C for 5 min.

#### Ovar DQA2

Primers DQA2s-up and DQA2s-dn (Hickford et al. 2004a) were used to amplify the exon 2 region of the *DQA2* locus. Each PCR reaction was carried out in a final volume of 20 μl containing 50 ng of genomic DNA, 0.25 μM of each primer, 250 μM dNTP (Invitrogen), 1 unit Taq DNA polymerase (Qiagen) and 1× reaction buffer containing 1.5 mM MgCl_2_ (supplied). Amplification consisted of denaturation at 94 °C for 2 min followed by 33 cycles of 94 °C for 30 s, 63 °C for 30 s and 72 °C for 45 s followed by an extension step at 72 °C for 5 min.

#### Ovar DQB1

Primers JM05 and JM06 (Table 1) were used to amplify the exon 2 region of the *DQB1* locus (Feichtlbauer-Huber et al. 2000). In addition, primer pair Lfl#991 and Lfl#994 (Atlija et al. 2015) were used to amplify the complete exon 2. Each PCR reaction was carried out in a final volume of 20 μl containing 50 ng of genomic DNA, 0.5 μM of each primer, 250 μM dNTP (Invitrogen), 1 unit Taq DNA polymerase (Qiagen), 1× reaction buffer (supplied) and 3.375 mM MgCl_2_. Amplification with primer pair JM05 and JM06 consisted of denaturation at 94 °C for 7 min followed by 33 cycles of 94 °C for 30 s, 60 °C for 30 s and 72 °C for 45 s followed by an extension step at 72 °C for 5 min. PCR amplification with Lfl#991 and Lfl#994 was carried out with a drop-down protocol for 15 min at 95 °C and 7× (95 °C for 30 s, 66–0.5 °C for 45 s, 74 °C for 45 s) and 30× (95 °C for 30 s, 63 °C for 45 s, 74 °C for 45 s).

#### Ovar DQB2

Primers JM05 and JM07 (Table 1) were used to amplify the exon 2 region of the *DQB2* locus. In addition, a modified version of JM05 (JM05mjs; Table 1) was combined with JM07 to amplify alleles AJ238935 and AJ238946. Each PCR reaction was carried out in a final volume of 20 μl containing 50 ng of genomic DNA, 0.5 μM of each primer, 250 μM dNTP (Invitrogen), 1 unit Taq DNA polymerase (Qiagen), 1× reaction buffer (supplied) and 3.375 mM MgCl_2_. Amplification consisted of denaturation at 94 °C for 7 min followed by 33 cycles of 94 °C for 30 s, 65 °C for 30 s and 72 °C for 45 s followed by an extension step at 72 °C for 5 min. Primer set 1001/1004 (Atlija et al. 2015) was used to amplify *DQB2* sequences but amplified multiple sequences in our hands and was not used for typing.

Amplimers were visualised by electrophoresis in 1.5% Seakem LE agarose (BioWhittaker Molecular Applications, Rockland, ME) gels using 1× TBE buffer containing 0.1 μg/ml ethidium bromide. PCR products were purified using the Qia-quick PCR Purification Kit (Qiagen) as per the manufacturer’s instructions and sequenced using the Big Dye® Terminator v3.1 Cycle Sequencing Kit (Life Technologies). The reactions were run on an ABI 3130.

#### Cloning

Cloning was used to identify new alleles and to resolve unclear sequences. Purified PCR products were cloned into pCR®4-TOPO® plasmid vector (Invitrogen) and then transformed into One Shot Top10 chemically competent *Escherichia coli* as per the manufacturer’s instructions (Invitrogen). Clones were grown on agar plates under ampicillin selection. Sixteen independent clones of each target sequence were picked and sequenced with the M13 forward primer as per the manufacturer’s instructions.

#### Sequence analysis

DNA sequences were examined with CLC Genomics Workbench software v7.0 (CLC Bio, Qiagen) and allocated to one or two alleles by blast searching and manual matching. Sequence alignments were made with CLC genomics, and similarity matrices were created using Clustal Omega on the EBI website (http://www.ebi.ac.uk/Tools/msa/clustalo/). Sequences were named after the DNA sequence whether DNA or protein sequences were being examined. This was to avoid confusion. Haplotype frequencies were determined with the allele procedure in SAS (SAS Institute, Cary, NC). Haplotypes were determined from the known pedigrees.

## Results

There were 18 *DRB1* sequences amplified from the 235 Texel lambs. Figure S1 shows the amino acid sequences produced by translating exon 2. Sixteen of these sequences are denoted by their IPD numbers. The other two sequences are awaiting numbers. As is typical of MHC alleles, there is considerable variation amongst alleles. Of the 89 amino acids in exon 2, 27 are polymorphic. The most distinctive allele is 0901. The proportion of identical amino acids between this allele and the other alleles ranged from 74 to 80%. In contrast, no other comparison amongst the other 17 alleles fell below 80%.

There were seven sequences amplified with the *DQA1* primer sets. Some haplotypes consistently failed to amplify any detectable product. The absence of a detectable product was defined as a null allele. In addition, there was one DQA2-like sequence amplified. The eight sequences are listed in Table 2, and their sequences are shown in Fig. S2. About half of the amino acids were polymorphic. The DQA2-like sequence was the most dissimilar sequence with a mean percent similarity of 62%, which indicates that over one third of the amino acids differed on average from the other proteins. This DQA2-like sequence was also one amino acid shorter than the other *DQA1* sequences.

**Table 2:** The class II haplotypes in a flock of Texel sheep

	*DRB1*	*DQA1*	*DQB1*	*DQA2*	DQA2-like	*DQB2*	Freq
1	0901	Z28518	LN868261	AY312388	NULL	AJ238939	0.0660
2	0601	M33304	NULL	AY312377	NULL	AJ238935 + AH001247	0.0340
3	0401	Z28418	Z28423	AY312381	NULL	HQ728669	0.0106
4	1601	AF276956	LN868260	AY312381	NULL	GU191456	0.1255
5	1501	AY265308	U07032	AY312386	NULL	LN811403	0.0085
6	1202	LN827890	HM367630	AY312387	NULL	GU191453	0.0064
7	0201	M33304	GU191455	AY312375	AY312392	GU191459 + U07033	0.0128
8	0805	Z28518	Z28424+ GU191455	AY312382	NULL	GU191459	0.0021
9	2202	LN736359	LN868258	AY312389	NULL	AJ238945	0.0128
10	2202	M33304	LN868258	AY312377	NULL	ABV90470	0.0043
11a	1101	NULL	NULL	AY312375	AY312392	AJ238935	0.1489
11b	1101	NULL	NULL	AY312375	AY312392	AJ238946	0.0532
12	1401	LN827890	HM367631	AY312387	NULL	GU191460	0.0787
13	0102	M33304	LN868259	AY312377	NULL	ABV90470	0.1298
14	HQ515541	M33304	LN868264	AY312377	NULL	AH001247	0.0106
15	0302	LN736359	LN868258	AY312389	NULL	AJ238945	0.0404
16	1001	M33304	GU191455	AY312382	NULL	GU191459	0.2106
17	0701	NULL	AJ238943	AY312375	AY312392	LN811404	0.0128
18	2002	M33304	LN868259	AY312377	NULL	ABV90470	0.0213
19	FM998807	M33304	LN868259	AY312377	NULL	ABV90470	0.0085
20	1001	LN827890	AJ238934	AY312387	NULL	GU191457	0.0021

The numbers for *DRB1* alleles refer to the allele numbers at the Immuno Polymorphism Database (IPD)

Eight alleles were amplified by the *DQA2* primer sets (Fig. S2). There was no null allele at *DQA2*. Almost one half of the amino acid sites in exon 2 were polymorphic (37/82). Although some pairs of alleles were quite similar (AY312375 and AY312377 as well as AY312381 and AY312382), the number of shared amino acids amongst alleles was quite low. After also excluding AY312388 and AY312389 (88% amino acids were identical), no other pair of alleles shared more than 80% of their amino acids. In other words, each allele differed from most other alleles in at least one fifth of its amino acid sites.

For *DQB1*, the primer sets amplified different sets of sequences. JM05 and JM06 (Feichtlbauer-Huber et al. 2000) amplified one set of sequences, whilst 991 and 994 (Atlija et al. 2015) amplified an overlapping but distinct set of sequences. Primers JM05 and 06 amplify part of the exon and may therefore be affected by gene conversion. Both primer sets did not simply amplify allelic series. Primer set JM05 and JM06 amplified three sequences from some animals. In each case, two of the sequences corresponded to GU191455 and GU191459. There were no instances of either sequence being amplified by JM05/JM06 without the other sequence. In contrast, the primer pair 991/994 amplified only GU191455 and not GU191459. Although, in some animals with GU191455, the primer set amplified the additional sequence Z28424. These animals with Z28424 differed in their *DRB1*, *DQA1*, *DQA2* and *DQB2* alleles, suggesting that the amplification of the additional sequence was unique to a specific haplotype. However, the primer set 991/994 sometimes only amplified one sequence in those animals where the primer set JM05/06 amplified two alleles; this simplified the typing of alleles but complicated the assignment of haplotypes. Figure S3 shows all the DQB sequences; those amplified by the primer set 991/994 have been listed as *DQB1* alleles in Table 2. We only tested one animal with the *DRB1* allele 0701, but the primer set 991/994 did not amplify any of the DQB sequences associated with this allele. There was also a null allele where there appeared to be no amplifiable sequence with both JM05/JM06 and 991/994. Both primer sets gave null alleles for haplotypes 11a and 11b.

The remaining 15 DQB alleles were not amplified by the primer set 991/994 and have provisionally been called *DQB2* alleles (Fig. S3). Some of these alleles were amplified by JM05/JM06 and some by JM06/07 or slightly modified versions of these primer sets. These primer pairs did not allow us to determine the first three amino acids or the last six amino acids of exon 2. The nine missing amino acids have been determined in other animals for some of these alleles, but we have only listed the complete exon 2 sequence for allele ABV 90470. Clearly, more work is necessary to determine the complete exon 2 sequence for this locus. Nonetheless, there is sufficient information to show that this locus is very polymorphic with 32 of the 80 known amino acid sites polymorphic in this population.

There were only 21 haplotypes in this population (Table 2). The number of possible haplotypes can be obtained by multiplying the number of alleles at each locus. Not all the DQ genes can be assigned to specific loci, but if we make the parsimonious assumption that there are 18 alleles at *DRB1*, 9 at *DQA1*, 13 at *DQB1*, 8 at *DQA2* and 16 at *DQB2*, there are 269,568 possible combinations of these 5 loci. As we only tested 235 animals (470 haplotypes) and many of the animals are related, the maximum observable number will be less than 470. Nevertheless, the small number of haplotypes was unexpected. There was also considerable variation in frequency. The most common haplotype occurred in 99 lambs, whilst the least common haplotype was present in only 1 lamb. Thirty sheep appeared homozygous for all loci in the class II haplotype. The homozygous haplotypes were 1 (2 sheep), 2 (1 sheep), 3 (5), 4 (5), 11a (4), 11b (1), 13 (4), 15(1) and 16 (7 sheep).

## Discussion

The examination of class II loci in a population of British Texel sheep has revealed considerable complexity and diversity amongst animals. There were 18 alleles at *DRB1*, but 3 of the 4 DQ loci were characterised by the absence of amplifiable products (null alleles) in some haplotypes. Two genes were amplified by the same set of primers in other haplotypes. Presumably, the absence of alleles and the existence of pseudo-alleles reflect the ongoing expansion and contraction of the DQ loci. This made the assignment of alleles to loci difficult. Different primer sets amplified distinct alleles, and this too complicated the assignment of alleles to loci. Applying the principles of parsimony, the simplest interpretation was that there were 9 alleles at *DQA1*, 13 alleles at *DQB1*, 8 alleles at *DQA2* and 16 alleles at *DQB2*. The definitive assignment of alleles to loci and loci to haplotypes may require complete sequencing of the MHC in relevant haplotypes.

There were eight alleles at *DQA1* including the null allele. We also assigned the DQA2-like molecule AJ312392 to *DQA1*. In three haplotypes, the DQA2-like gene occurred when there was no classical *DQA1* allele. This confirms previous results from New Zealand sheep (Hickford et al. 2004a) and supports the assignment of this molecule to the *DQA1* locus. However, haplotype 7 carried both a *DQA1* allele and a DQA2-like gene. This haplotype also carried two *DQB1* genes. The DQA2-like genes contain elements of *DQA1* and *DQA2* genes and probably arise by intergenic recombination (gene conversion) (Ballingall et al. 2015; Hickford et al. 2004a). This process creates new alleles at existing loci and is not expected to create new loci. The extra loci on haplotype 7 are more likely to have arisen by a gene duplication event involving both *DQA1* and *DQB1* loci. This duplication could have been followed by a gene conversion event to create the DQA2-like gene.

There were no null alleles and no duplicated alleles at the *DQA2* locus. This locus therefore had the simplest structure of the DQ loci and with only eight alleles; it had fewer alleles than the other class II antigen-presenting loci. In some ways, it is the least diverse of the classical class II loci. However, there were large differences amongst alleles, and whether the DQA loci are more or less diverse than the DQB loci depends upon the criterion; counting alleles gives a different result than counting amino acid differences between alleles.

Different haplotypes encoded 0, 1 or 2 *DQB1* sequences. The 11a and 11b haplotypes did not code for amplifiable sequences, but these haplotypes were quite common at 15 and 5% of the population. In three spined stickleback fish, individuals with intermediate numbers of MHC class IIB genes are more resistant to parasite infection (Wegner et al. 2003), but similar analyses have yet to be performed in sheep.

We did not amplify the complete exon 2 sequence for the *DQB2* alleles. A more comprehensive definition of class II variation would also include the complete protein coding regions as well as polymorphisms in promoter regions and additional regulatory elements. In addition, in sheep, the DRA gene is polymorphic (Ballingall et al. 2010), and it would be useful to test this gene.

Although there were a relatively large number of alleles defined at the DR and DQ loci, these alleles produced relatively few haplotypes. The number of alleles and haplotypes in a population will be determined by the processes of mutation and gene conversion followed by selection and drift. Drift will cause the loss of genetic variation, and the amount of loss will depend upon the effective population size. Our population was relatively small, and like all farm animal populations, many of the individuals were related to each other. Countering the losses due to drift, the British Texel population was formed by grading up from British sheep, and our population was formed by grading up a Texel-Oxford population. These ancestral populations may have contributed some of the alleles and haplotypes. In addition, some of the rams came from other flocks, and this would have increased the effective size of the population. The class II region in sheep is relatively compact, and the DRB and DQ loci occur in less than 160,000 bases (Herrman-Hoesing et al. 2008). This relatively small distance would reduce the rate of recombination and slow the formation of new haplotypes. We did not observe any recombination in our animals, although recombination within the class II region does occur (Hickford et al. 2007). The existence of relatively few haplotypes within a breed will make it easier to define associations between the MHC and diseases (Stear et al. 2007) but more difficult to identify the causative mutations.

In summary, we have defined alleles and haplotypes in a population of British Texel sheep by sequencing PCR products. Exon 2 variation defined 18 *DRB1* alleles and demonstrated a complex pattern of variation in the DQ genes. Most haplotypes had one allele at each locus, but some loci had no identifiable product whilst other putative loci had two PCR products, which suggest gene duplication. Often but not always, the DQA and DQB genes formed pairs. This complexity adds to the diversity of the MHC region but complicates the identification of causative mutations within the MHC that determine variation to disease resistance.

## Electronic supplementary material

Figure S1 The amino acid sequence of exon 2 of *DRB1* alleles in Texel sheep. The numbers for *DRB1* alleles refer to the allele numbers at the Immunopolymorphism database (IPD). (JPEG 6031 kb)

Figure S2 The amino acid sequence of exon 2 of *DQA1* and *DQA2* alleles in Texel sheep. The allele names refer to the designations in the Genbank database. Alleles at both loci have been listed together to simplify sequence comparison. (JPEG 6303 kb)

Figure S3 The amino acid sequence of exon 2 of *DQB1*and *DQB2* alleles in Texel sheep. The allele names refer to the designations in the Genbank database. Alleles at both loci have been listed together for comparison. (JPEG 7171 kb)
